# Vertical and seasonal changes in soil carbon pools to vegetation degradation in a wet meadow on the Qinghai-Tibet Plateau

**DOI:** 10.1038/s41598-021-90543-6

**Published:** 2021-06-10

**Authors:** Jiangqi Wu, Haiyan Wang, Guang Li, Jianghua Wu, Weiwei Ma

**Affiliations:** 1 College of Forestry, Gansu Agricultural University, Lanzhou, 730070 China; 2 School of Science and the Environment, Memorial University of Newfoundland, 20 University Drive, Corner Brook, NL A2H 5G4 Canada

**Keywords:** Ecology, Environmental sciences

## Abstract

Wet meadows provide opportunities to decrease carbon dioxide (CO_2_) and methane (CH_4_) released into the atmosphere by increasing the soil organic carbon (SOC) stored in wetland systems. Although wet meadows serve as the most important and stable C sinks, there has been very few investigations on the seasonal distributions of SOC fractions in high-altitude wet meadows. Here, we studied the effects of four vegetation degradation levels, non-degraded (ND), lightly degraded (LD), moderately degraded (MD), and heavily degraded (HD), on the measured vertical and seasonal changes of SOC and its different fractions. Among these vegetation degradation levels, 0–10 and 10–20 cm soil depths in ND plots had significantly higher SOC contents than the other degradation levels had throughout the year. This is attributed to the relatively greater inputs of aboveground plant litter and richer fine-root biomass in ND plots. Particulate organic carbon (POC) and light fraction organic carbon (LFOC) showed similar vertical and seasonal variations in autumn, reaching a minimum. Moreover, microbial biomass (MBC) and easily oxidizable organic carbon (EOC) contents were highest in summer and the smallest in winter, while dissolved organic carbon (DOC) content was highest in spring and lowest in summer, and were mainly concentrated in the 0–20 cm layer. Pearson correlation analysis indicated that soil properties and aboveground biomass were significantly related to different SOC fractions. The results indicate that vegetation degradation reduces the accumulation of total SOC and its different fractions, which may reduce carbon sink capacity and soil quality of alpine wet meadows, and increase atmospheric environmental pressure. In addition, vegetation biomass and soil characteristics play a key role in the formation and transformation of soil carbon. These results strengthen our understanding of soil C dynamics, specifically related to the different C fractions as affected by vegetation degradation levels and soil depth, in wet meadow systems.

## Introduction

Wetlands are important for organic carbon storage, and small changes will affect carbon content in the atmosphere^1^. Wetland ecosystems play a role that other ecosystems cannot replace in improving the climate and maintaining regional ecological balance^2^. However, with global warming and intensified human development (such as overgrazing), wetland areas have shrunk and groundwater levels havelowered^3,4^, causing changes in vegetation composition and thus, in some cases, vegetation degradation. In addition, previous studies on peatlands, floodplain wetlands, plateau wetlands and coastal wetlands found that vegetation degradation in wetlands reduces species diversity and net primary productivity, significantly reducing the functions of wetlands as carbon sinks and total carbon storage^1,5–7^.

Soil plays an important role in ecosystem carbon cycling^8^. For example, small changes in soil carbon pools will affect the global carbon cycle and atmospheric carbon concentration^9^. Soil organic carbon (SOC) is a complex organic heterogeneous body^10^, and changes in SOC cannot directly and quickly reflect changes in soil quality^11^, while some labile organic C fractions, including microbial biomass C (MBC), dissolved organic C (DOC), light fraction organic C (LFOC), easily oxidizable organic C (EOC), and particulate organic C (POC), are sensitive to and respond rapidly to soil quality under different conditions^12,13^. Therefore, changes in different soil organic carbon fractions are considered as early indicators of changes in soil quality and carbon storage^14^. A study in the Loess Plateau in China found that the restoration of vegetation from 1998 to 2006 resulted in an increase of 19% SOC reserves in the 0–20 cm layer^15^. Wang et al.^16^ found that when the vegetation cover of alpine meadows was reduced from 80 to 15 dm^2^/m^2^, the topsoil’s LFOC content decreased by 38.4–86.7%. In addition, due to the complex and comprehensive effects of various ecological factors (climate, soil, vegetation) and different dominant positions of key factors, significant differences in soil environment, nutrient supply and microbial activities have led to changes in soil organic carbon composition^17,18^. Eventually, this leads to differences in the seasonal variation in patterns of soil active organic carbon content in wetland ecosystems^19–21^. Thus, the seasonal response of the different components of SOC to vegetation degradation is a more effective approach to predict the effects of vegetation degradation on SOC pool dynamics in wet meadow ecosystems. However, knowledge concerning the variation in the different fractions of SOC under different levels of vegetation degradation is poor.

The Qinghai-Tibet Plateau (QTP) occupies about a quarter of China's land area and is a key area for China's water resources and ecological security^22^. The area of wet meadow accounted for 35% of QTP^23^. In recent years, climate change here has been more significant and advanced compared with other areas^24^. That is to say, the mean annual temperature in the QTP has increased at a rate of 0.16 °C per decade^25^, and at the same time grazing intensity has exceeded the theoretical grazing capacity of the ecosystem^26^, which results in the wet meadows in the QTP also experiencing largescale vegetation degradation, shrinkage and transformation^27^. Succession in the wetland plant communities has changed from wet meadows to grassland and sandy meadows^25^. At present, most studies about soil C dynamics in the QTP have mainly focused on the impact of permafrost, wetland drainage, and climate change (temperature and rainfall) on total SOC content^28–31^. In addition, Rui et al.^32^ studied the effects of warming and grazing on the contents of DOC and MBC. Previous studies have shown that vegetation degradation in the alpine meadow increases soil C emission to the atmosphere and thus global warming potential^33^, and reduces soil MBC, DOC and SOC contents^34,35^. Moreover, perennial plants participate in SOC formation by providing organic C via rhizosphere exudates and litter, which have a positively influences SOC^17^. The different components of SOC are very sensitive to time, and seasonal changes may play a vital role in nutrient supply and microbial activity. For example, during the growing season of the region’s vegetation (May to September), we found that the seasonal trends of soil LFOC and DOC were not consistent^36^. Not only is the growing season important to wet meadows in this region, but the spring–autumn-winter period is also critical to their ecosystem function and soil C cycling because plant growth is slow or non-existent, and lower soil temperatures affects microbial activity and the ability to decompose litter^37^. During these periods, frequent temperature changes will affect processes of SOC conversion in the C cycle^38,39^. However, little is known about how vegetation degradation in wet meadows of the QTP affects the seasonal changes of SOC components in the non-growing season (spring, autumn, and winter). Therefore, in order to better understand the SOC transformation processes in the Tibetan Plateau, changes in the SOC cycle under different vegetation degradation levels during the non-growing season in the Tibetan wet meadow require further investigation.

To address these knowledge gaps, this study assessed the seasonal responses of different soil carbon fractions to different degrees of vegetation degradation in a QTP wet meadow. We measured the contents different fractions of SOC (i.e., SOC, MBC, DOC, EOC, POC, and LFOC) during the four seasons of a year. The objectives of this study were to: (1) conduct a quantitative analysis of the effects of vegetation degradation on different SOC fractions; (2) study the seasonal dynamics of SOC’s different fractions due to vegetation degradation, and (3) determine correlations among SOC fractions and basic soil properties. We hypothesized that: (1) with increasing vegetation degradation intensity, the SOC content will decrease because of lower carbon source inputs and higher carbon decomposition after vegetation degradation; and (2) the patterns in seasonal variation of SOC components differ with different intensity of vegetation degradation due to the complexity of various ecological factors (climate, soil, vegetation) and the different dominant position of key factors.

## Materials and methods

### Study area

**Figure 1:**
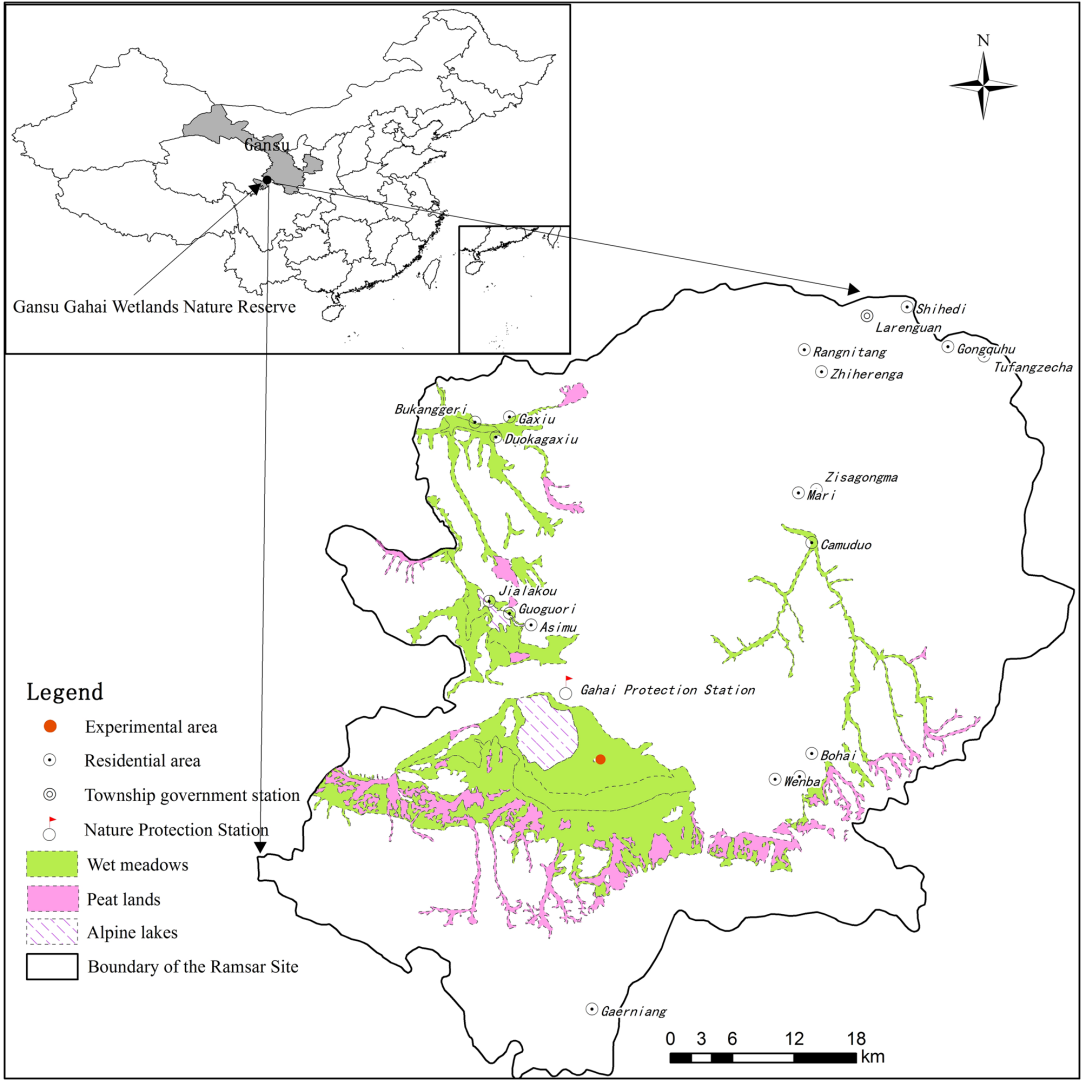
The geographical location map of the study area, the red dot represents the specific location after amplification^40^.

The study is located in Gahai-Zecha National Nature Reserve (33°58′12″–34°32′16″ N, 102°05′00″–102°47′39″ E), Luqu County, Gansu Province, China. The area altitude is 3430–4300 m, and the wet meadow area is 4.07 × 10^4^ ha, which is the main wetland type in the reserve (Fig. 1). This region belongs to the Qinghai-Tibet Plateau climatic zone (a cold temperate continental monsoon climate). The weather data from 1981 to 2020 showed that the average annual temperature in the area was 2.9 °C (http://data.cma.cn/data/weatherBk.html). The average temperature in July (12.9 °C) is 21.4 °C higher than that in January (− 8.5 °C). The mean annual precipitation was 785 mm, with 76.4% of this amount occurring during the May–September growing season.

### Experimental design and soil sampling

**Figure 2:**
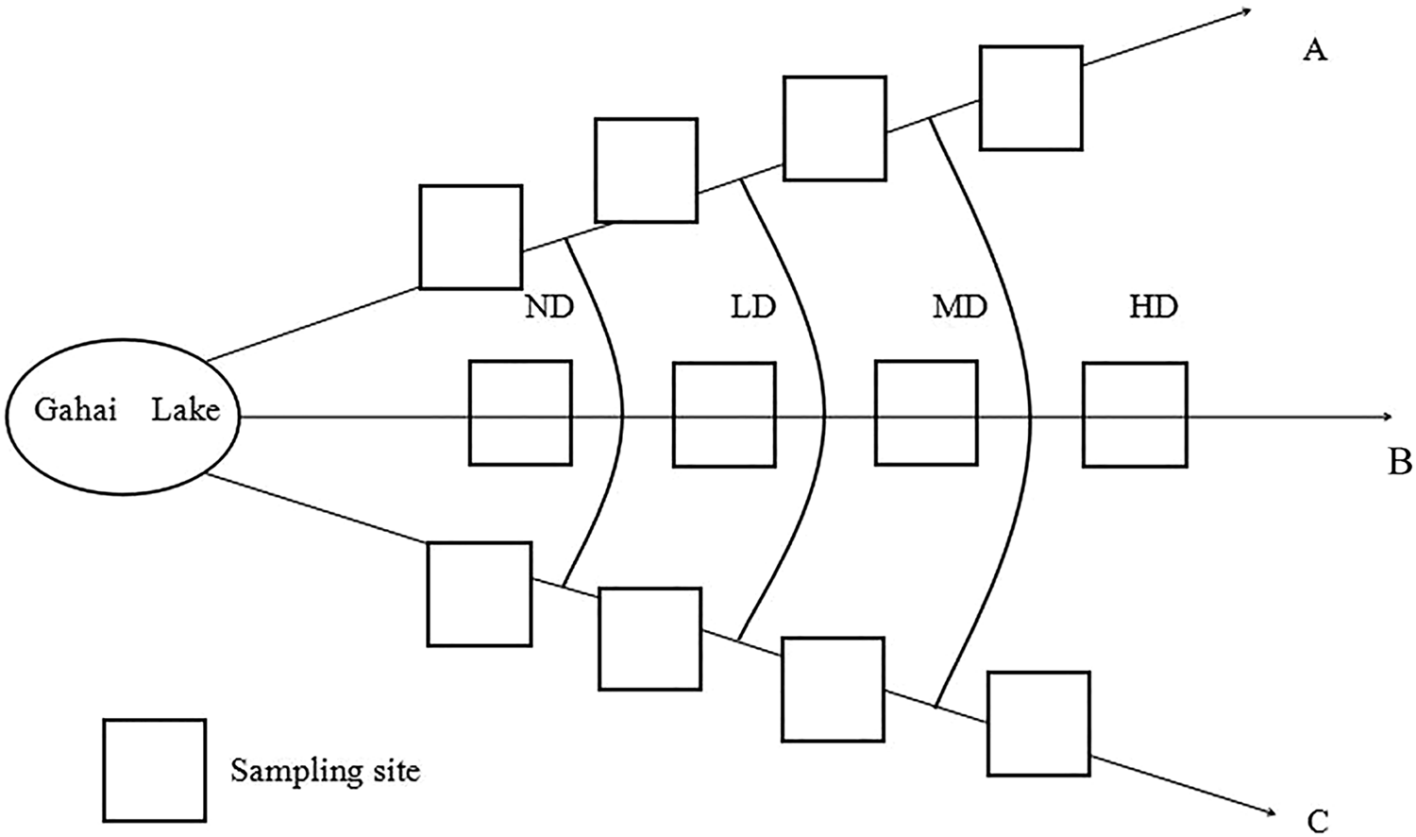
Distribution of sampling sites. A, B, and C represent four transects. *ND* non-degraded, *LD* lightly degraded, *MD* moderately degraded, *HD* heavily degraded.

In the 1950s, the grazing intensity exceeded the theoretical grazing capacity of the ecosystem, which led to the degradation of wetland vegetation^25^. We selected representative near-mountain lakes in the study area, and established three linear transects (A, B, and C) for the level of vegetation degradation in a radial pattern along a moisture gradient (Fig. 2). At the same time, vegetative cover, dominant species, and biomass were identified as key indicators for the assessment of wet meadow degradation levels^23,34^. Finally, we, in early May 2013, divided wet meadows of the three linear transects into four degradation levels based on vegetation species composition, aboveground biomass, vegetative cover, and groundwater level. The four wet meadow levels are non-degraded (ND), lightly degraded (LD), moderately degraded (MD), and heavily degraded (HD) (Table S1), and the area of each degraded sample plot was 10 m × 10 m. To reduce potential edge effects, we kept at least 5 m buffer zone between two linear transects. We collected soil samples in four different seasons: spring, summer, autumn, and winter^17,41^. Soil samples were collected in each of the twelve plots after removing dead leaves from the surface layer on 20th April, 18th July, 25th October 2018, and 25th January 2019. Samples were obtained by using an auger with a diameter of 50 mm at seven points in each plot (Fig. S1, the human in the Fig. S1 is the first author), for which two points were near opposite sides of the plot and three points along a diagonal across the plot, forming a “Z” pattern. Each of the seven samples collected in each plot was divided into six sampling intervals at different depth (i.e., 0–10, 10–20, 20–40, 40–60, 60–80, and 80–100 cm), resulting in 4 × 3 × 6 × 7 = 504 samples. In order to avoid cross-contamination of the soil between different soil layers, we only took the middle part of the soil column. For example, the diameter of the soil column taken out each time is 50 mm, and the height is 10 cm. We removed the upper and lower layers of the soil column (the upper and lower layers are each 3 cm thick), and only the middle 4 cm was used to make the mixed soil sample, and each sample was taken always after we cleaned the inside of the soil auger. 72 Samples (4 × 3 × 6 = 72) from the same soil depth interval were combined to form a mixed soil sample that was taken back to the laboratory for analysis. At the same time when the soil was collected, we used a portable digital thermometer (JM624, Jinming Instrument Co., Tianjin, China) to measure the soil temperature at 5 cm. The sharp-edged ring (7.6 cm diameter, 6.5 cm long) method was used to collect soil samples from each plot on July 18th, 2019 to determine the bulk density (BD).

### Soil analysis

We used gravimetric analysis to determine soil BD^42^. Briefly, we used the cutting ring to retrieve the original soil, dried the soil at 105 °C, measured the dry weight of the soil, and then calculated the soil BD. Soil pH was assayed from a water suspension with a 1:2.5 (W:V) soil–water ratio^43^ and measured by a pH meter (Consort C830). We used the Kjeldahl method to determine the total nitrogen (TN) content in the soil, and the molybdenum colorimetric method to determine the total phosphorus (TP)^44,45^. The basic soil properties were shown in Table 1 and Fig. S2.

**Table 1:** Bulk density and contents of the main soil nutrients (mean ± standard deviation, n = 3) in the 0–100 cm layer of the four-degradation level.

Treatment	Soil depth (cm)	BD (g cm^−3^)	TN (g kg^−1^)	TP(mg kg^−1^)	PH
ND	0–10	0.40 ± 0.01 C	6.30 ± 0.05 A	67.80 ± 1.33 A	7.94 ± 0.07 A
	10–20	0.43 ± 0.01 D	5.77 ± 0.20 A	63.34 ± 1.22 A	7.96 ± 0.04 A
	20–40	0.44 ± 0.01 D	5.17 ± 0.13 A	49.77 ± 1.08 B	7.90 ± 0.01 A
	40–60	0.46 ± 0.01 D	3.64 ± 0.03 A	58.61 ± 0.83 B	7.90 ± 0.01 A
	60–80	0.47 ± 0.01 D	3.34 ± 0.04 A	33.55 ± 0.50 D	–
	80–100	0.49 ± 0.01 D	2.58 ± 0.07 B	26.09 ± 1.22 C	–
LD	0–10	0.42 ± 0.01 C	5.90 ± 0.03 B	61.01 ± 1.74 B	7.69 ± 0.00 B
	10–20	0.48 ± 0.01 C	4.35 ± 0.10 B	43.95 ± 0.82 C	7.73 ± 0.01 B
	20–40	0.48 ± 0.01 C	4.02 ± 0.05 C	37.59 ± 3.14 D	7.81 ± 0.01 C
	40–60	0.48 ± 0.01 C	3.28 ± 0.04 C	44.60 ± 1.13 C	7.85 ± 0.01 BC
	60–80	0.50 ± 0.01 C	2.92 ± 0.05 C	43.22 ± 0.57 B	–
	80–100	0.51 ± 0.01 C	2.86 ± 0.07 A	32.89 ± 0.89 B	–
MD	0–10	0.54 ± 0.02 B	4.83 ± 0.10 C	58.87 ± 0.80 B	7.65 ± 0.02 B
	10–20	0.64 ± 0.01 B	4.12 ± 0.07 B	59.35 ± 0.99 B	7.75 ± 0.01 B
	20–40	0.65 ± 0.01 B	3.96 ± 0.05 C	42.89 ± 0.21 C	7.84 ± 0.01 B
	40–60	0.67 ± 0.01 B	3.50 ± 0.09 B	46.49 ± 1.44 C	7.86 ± 0.01 B
	60–80	0.69 ± 0.01 B	3.34 ± 0.05 A	37.18 ± 0.70 C	–
	80–100	0.71 ± 0.01 B	3.02 ± 0.15 A	31.57 ± 0.80 B	–
HD	0–10	0.64 ± 0.02 A	4.03 ± 0.07 D	52.59 ± 0.74 C	7.68 ± 0.01 B
	10–20	0.69 ± 0.02 A	3.37 ± 0.07 C	64.94 ± 0.83 A	7.75 ± 0.01 B
	20–40	0.70 ± 0.01 A	4.19 ± 0.05 B	53.83 ± 1.33 A	7.83 ± 0.00 B
	40–60	0.72 ± 0.01 A	3.41 ± 0.03 B	61.72 ± 1.52 A	7.84 ± 0.01 C
	60–80	0.73 ± 0.01 A	3.22 ± 0.08 B	44.52 ± 0.89 A	–
	80–100	0.76 ± 0.02 A	2.68 ± 0.02 B	35.72 ± 0.48 A	–

*BD* bulk density, *TN* total nitrogen, *TP* total phosphorus, *ND* non-degraded, *LD* lightly degraded, *MD* moderately degraded, *HD* heavily degraded. Different capital letters (A, B, C, and D) indicate significant differences among vegetation degradation levels for the same soil layer and season based on Duncan's multiple comparison test at P < 0.05.

We determined the SOC content according to the method by Wang et al.^46^. Briefly, the soil sample (0.1 g, accurate to 0.001 g) was extracted with 7.5 ml of 0.4 M of K_2_Cr_2_O_7_ and 7.5 ml of concentrated H_2_SO_4_ at 180 °C for 30 min, and then calculate the SOC content based on the consumption of potassium dichromate^35^.

Fresh soil samples equivalent to 10 g dry soil were weighed and poured into a 150 ml triangular bottle, and 50 ml 0.5 M K_2_SO_4_ solution was added and allowed to shake for 1 h. This solution was then centrifuged for 10 min at 3000 R min^−1^^17^. We draw 5 ml of supernatant to determine DOC content according to the method by Li et al.^47^.

The MBC was determined using fresh soil samples according to Nie et al^48^. The fumigated and non-fumigated soil (sieving < 2 mm) were extracted with 0.5 M K_2_SO_4_. 5 ml supernatant was extracted and titrated according to the method of organic carbon. In addition, we used the method by Shao et al.^21^ to calculate the MBC content: (C_fumigated_ – C_non-fumigated_)/0.38.

We put 2 g soil into a 50 ml centrifuge tube, added 25 ml of 0.333 M KMnO_4_ solution, shaken for 1 h (180 r min^−1^) and then centrifuged for 5 min (5000 r min^−1^). 1 ml of the supernatant was diluted 250 folds and absorbance at 565 nm was determined. The blank control group was added with the same amount of solution (just no soil). Calculate the EOC content based on the difference between the sample and the blank control group^14,49^.

We determined the POC content according to the method described by dos Reis Ferreira et al.^50^. Briefly, we added 100 ml (NaPO_3_)_6_ solution (5 g L^−1^) to 20 g of soil and placed it on a reversible shaker (180 r min^−1^) for 16 h. We repeatedly rinsed the soil on the 53 μm sieve with distilled water, and weighed the remaining soil on the sieve after dried at 60 °C. Then we weighed 0.01 g of soil to determine the organic carbon content^51^.

We determined the LFOC content according to the Li et al.^52^ method. Briefly, we added 10 ml of NaI solution (density 1.7 µg ml^−1^) to 5 g soil and shaked for 60 min. After centrifugation, we passed the supernatant through a Millipore filter and collected the light fractions, again extracted the soil residue in the centrifuge with NaI, and collected additional light fraction fractions. We combined the two light fraction components, dried at 60 °C, and sieved through 60 mesh sieve. Using 0.02 g of light fraction components, the soil organic carbon content was determined according to the method by Luan et al.^51^.

### Statistical analysis

The significance of the differences in soil carbon components and basic soil properties (i.e., BD; TN; pH; and TP) among different treatments was analyzed using Duncan’s analysis of variance in SPSS 19.0 software (significance level of 95%, P < 0.05). A repeated-measures ANOVA was employed to determine differences in SOC, EOC, POC, LFOC, MBC, and DOC among vegetation degradation levels using the season and soil depth as the repeated variable. Pearson’s correlation coefficients were derived between soil SOC fractions and basic soil properties, aboveground biomass with an accepted significance level of P < 0.05. Moreover, linear regression analysis was used to explore the relationship between soil temperature and POC, MBC, and EOC.

## Results

### SOC contents across vegetation degradation levels and soil depths

**Figure 3:**
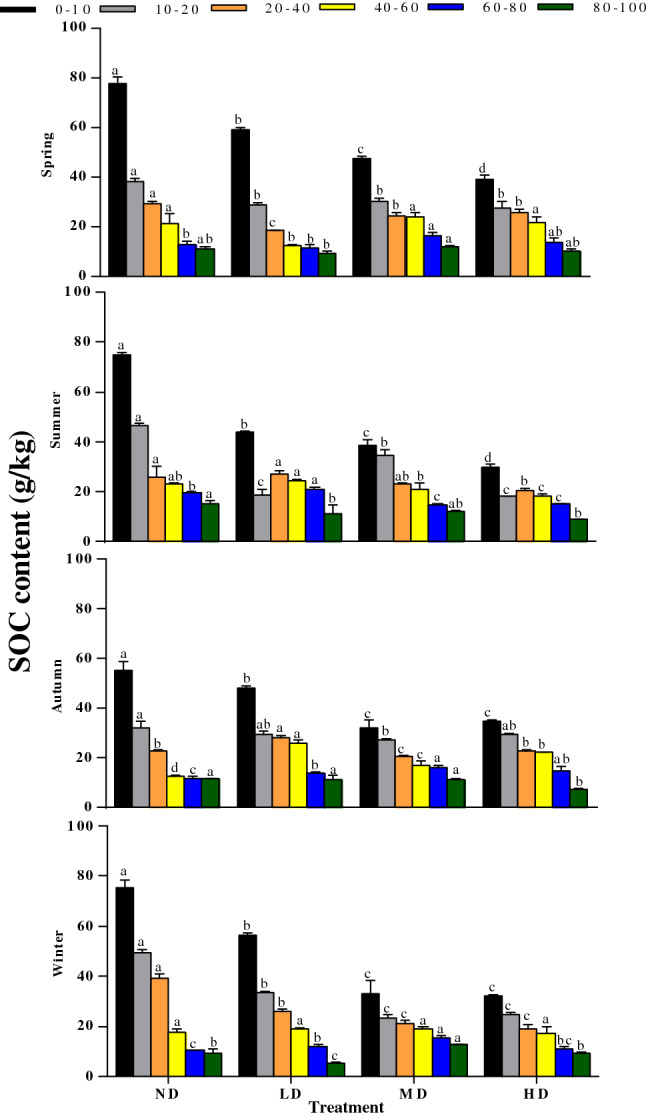
Seasonal variations in SOC at different depths (0–10, 10–20, 20–40, 40–60, 60–80, and 80–100 cm) in different vegetation degradation levels. *ND* non-degraded, *LD* lightly degraded, *MD* moderately degraded, *HD* heavily degraded. Error bars indicate standard errors of the mean (n = 3). Different lowercase letters indicate significant differences among vegetation degradation levels for the same soil layer and season based on Duncan's multiple comparison test at P < 0.05.

As the soil depth increased, SOC changed greatly in the four vegetation degradation levels (Fig. 3). The SOC content was significantly higher in the upper two soil layers (0–10 and 10–20 cm) but was relatively low in the bottom four layers (P < 0.05). The SOC contents in the 0–10 and 10–20 cm layers of the ND plot was significantly higher than those in the other vegetation degradation plots throughout the year (P < 0.05). The average SOC content of the four seasons in the ND area was significantly higher than that in the other three degradation levels (P < 0.05). In the ND plot, the mean SOC content in summer was 8.28%, 39.88%, and 1.81% higher than that in spring, autumn, and winter, respectively. The highest mean SOC content for each season was recorded from the ND plot (31.62, 34.24 and 33.63 g kg^−1^ in spring, summer and winter, respectively). The repeated-measures ANOVA showed significant interactions between season and vegetation degradation on SOC in all sample layers (Table 2).

**Table 2:** Results of a repeated-measures ANOVA testing for differences in soil organic carbon components (MBC, DOC, EOC, POC, LFOC, SOC) among planting systems using season and depth as the repeated variable.

Source of variation	df	MBC		DOC		EOC		POC		LFOC		SOC	
		F	P	F	P	F	P	F	P	F	P	F	P
VD	3	17.181	0.000	126.870	0.000	137.618	0.000	364.911	0.000	484.268	0.000	446.922	0.000
S	3	335.549	0.000	35.003	0.000	353.104	0.000	143.381	0.000	26.189	0.000	21.524	0.000
D	5	231.357	0.000	583.366	0.000	915.146	0.000	1496.203	0.000	1619.621	0.000	2620.202	0.000
VD × S	9	5.606	0.000	58.564	0.000	28.478	0.000	14.845	0.000	16.665	0.000	52.836	0.000
VD × S × D	45	3.427	0.000	26.797	0.000	7.140	0.000	6.156	0.000	7.182	0.000	14.740	0.000

*VD* vegetation degradation, *S* season, *D* depth.

### LFOC and POC contents across vegetation degradation levels

**Figure 4:**
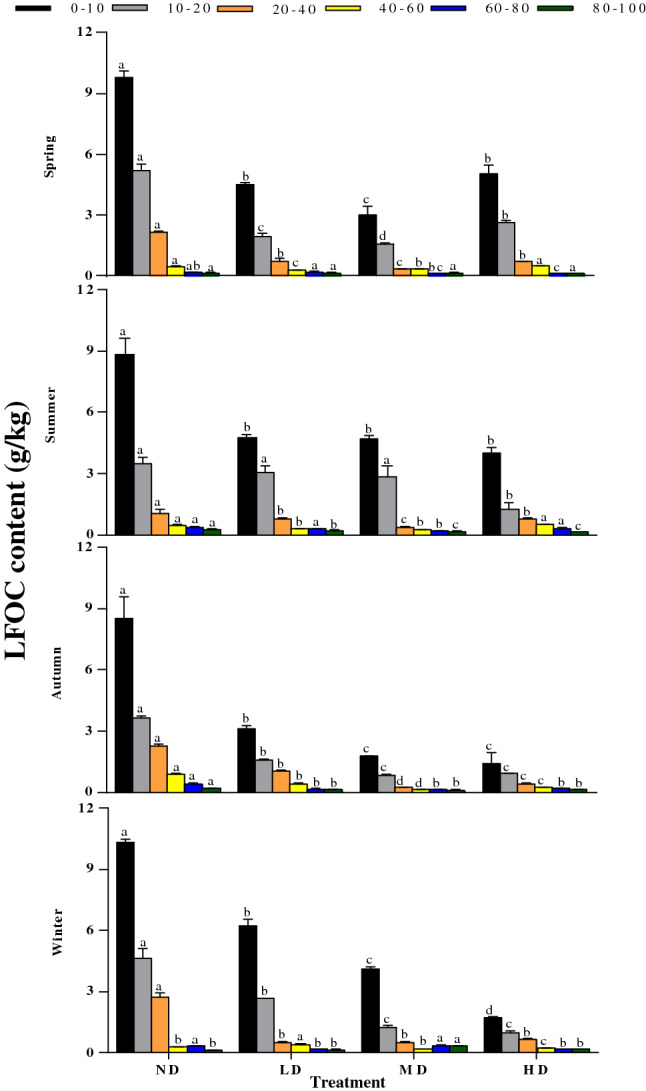
Seasonal variations in LFOC at different depths (0–10, 10–20, 20–40, 40–60, 60–80, and 80–100 cm) in different vegetation degradation levels. *ND* non-degraded, *LD* lightly degraded, *MD* moderately degraded, *HD* heavily degraded. Error bars indicate standard errors of the mean (n = 3). Different lowercase letters indicate significant differences among vegetation degradation levels for the same soil layer and season based on Duncan's multiple comparison test at P < 0.05.

**Figure 5:**
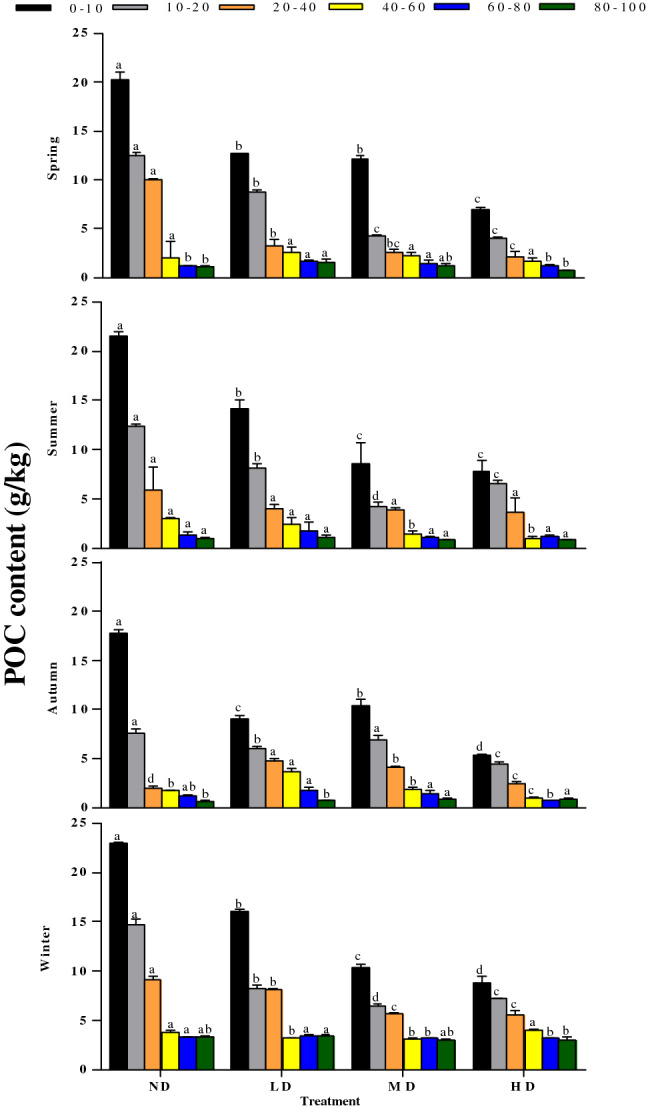
Seasonal variations in POC at different depths (0–10, 10–20, 20–40, 40–60, 60–80, and 80–100 cm) in different vegetation degradation levels. *ND* non-degraded, *LD* lightly degraded, *MD* moderately degraded, *HD* heavily degraded. Error bars indicate standard errors of the mean (n = 3). Different lowercase letters indicate significant differences among vegetation degradation levels for the same soil layer and season based on Duncan's multiple comparison test at P < 0.05.

Soil LFOC and POC belong to the physically uncomplexed type of organic matter. Their organic carbon content were significantly different from the ND to the HD areas (Figs. 4 and 5). The average LFOC and POC contents gradually decreased with the deterioration of vegetation degradation, except for the slight increase in LFOC content in the HD area. The LFOC and POC contents decreased with the increase in soil depth during the four seasons. The LFOC and POC contents were significantly greater in the upper two soil layers (0–10 and 10–20 cm) than in the bottom four layers (P < 0.05). The LFOC contents in the 0–10 and 10–20 cm layers of the ND area were significantly greater than in the other degradation levels throughout the year (P < 0.05). However, no significant differences were observed in the 20–100 cm layers throughout the year except in winter (P > 0.05). In the ND plot, the mean LFOC content in winter was 2.66%, 26.93%, and 15.74% higher than that in spring, summer, and autumn, respectively. However, the highest mean POC content observed among the four seasons was in winter, 9.54, 7.08, 5.32, and 5.27 g kg^−1^ in ND, LD, MD, and HD, respectively. The repeated-measures ANOVA showed significant interactions between season and vegetation degradation on LFOC and POC in all sample layers (Table 2).

### Soil active organic C content across vegetation degradation levels

**Figure 6:**
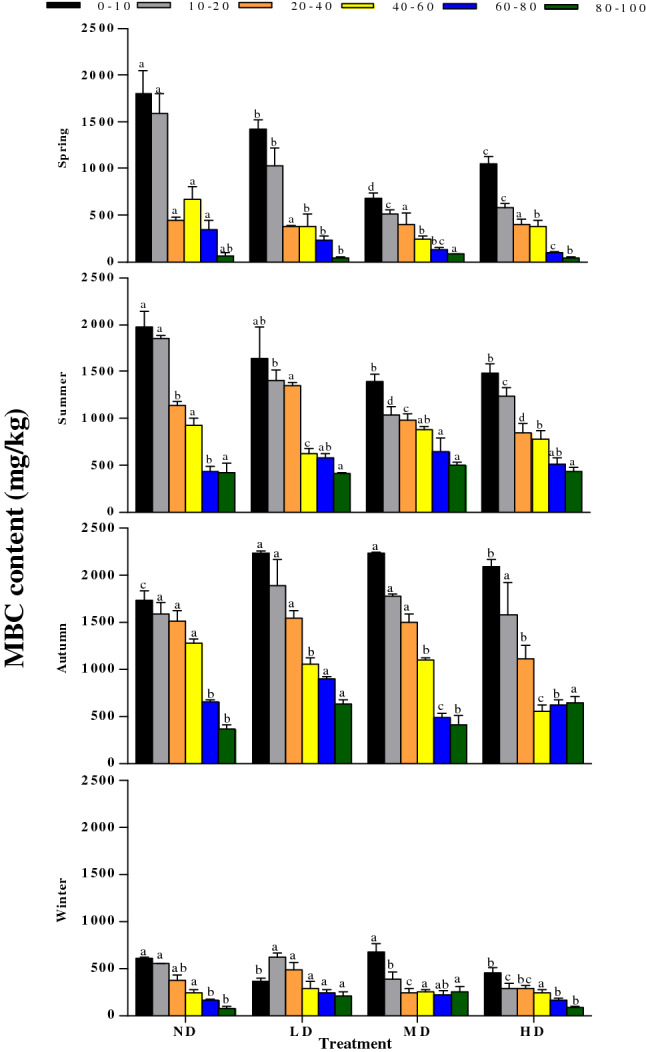
Seasonal variations in MBC at different depths (0–10, 10–20, 20–40, 40–60, 60–80, and 80–100 cm) in different vegetation degradation levels. *ND* non-degraded, *LD* lightly degraded, *MD* moderately degraded, *HD* heavily degraded. Error bars indicate standard errors of the mean (n = 3). Different lowercase letters indicate significant differences among vegetation degradation levels for the same soil layer and season based on Duncan's multiple comparison test at P < 0.05.

Soil MBC, DOC and EOC are important indicators of soil active organic C. In the four different levels of degradation, the highest value of MBC concentration was found in soil depth of 0–100 cm in the ND region (mean = 747.05 mg kg^−1^) followed by that in the LD region (mean = 730.93 mg kg^−1^), and the lowest was in the HD region (mean = 577.59 mg kg^−1^) (Fig. 6). Furthermore, significant seasonal changes were observed in soil MBC contents among the four treatments (P < 0.01). The average content of soil MBC was highest in autumn, followed by spring and summer, and lowest in winter. The MBC content in the top soil layers (0–10 and 10–20 cm) of the ND treatment was significantly higher than those of other treatments in spring and summer (P < 0.05), while contents in the same soil layers of the ND treatment were significantly lower than those of the other treatments in autumn (P < 0.05). In winter, MBC contents of the four degraded levels differed slightly in winter.

**Figure 7:**
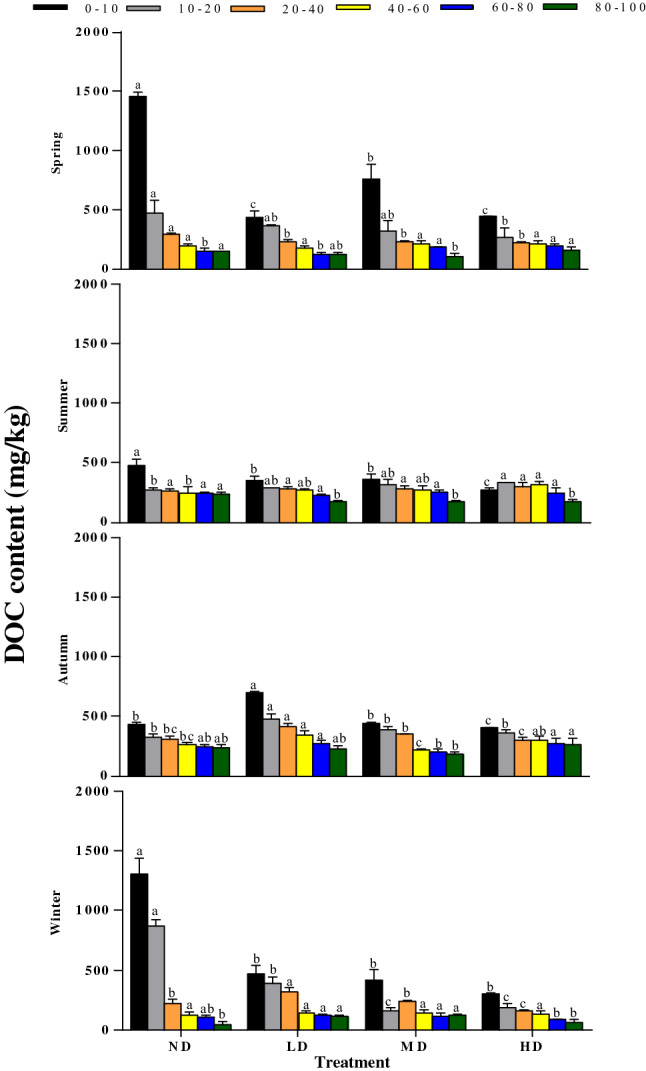
Seasonal variations in DOC at different depths (0–10, 10–20, 20–40, 40–60, 60–80, and 80–100 cm) in different vegetation degradation levels. *ND* non-degraded, *LD* lightly degraded, *MD* moderately degraded, *HD* heavily degraded. Error bars indicate standard errors of the mean (n = 3). Different lowercase letters indicate significant differences among vegetation degradation levels for the same soil layer and season based on Duncan's multiple comparison test at P < 0.05.

**Figure 8:**
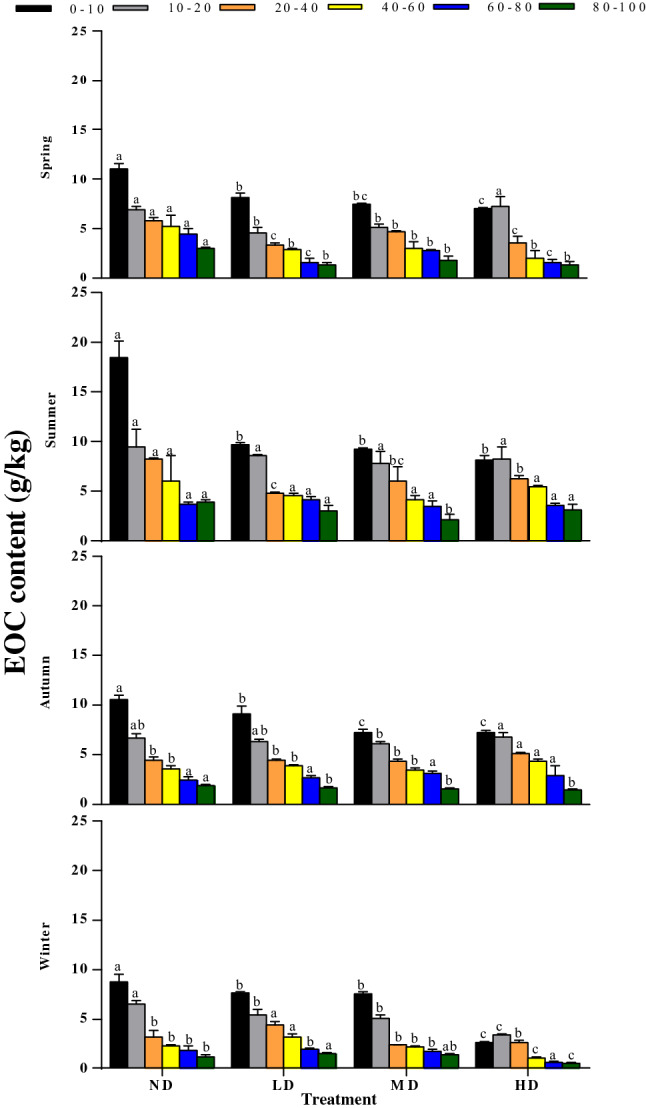
Seasonal variations in EOC at different depths (0–10, 10–20, 20–40, 40–60, 60–80, and 80–100 cm) in different vegetation degradation levels. *ND* non-degraded, *LD* lightly degraded, *MD* moderately degraded, *HD* heavily degraded. Error bars indicate standard errors of the mean (n = 3). Different lowercase letters indicate significant differences among vegetation degradation levels for the same soil layer and season based on Duncan's multiple comparison test at P < 0.05.

Wet meadow DOC concentration declined gradually within increasing degradation level from ND to HD (Fig. 7). At the 0–10 and 10–20 cm layers, the contents of DOC in ND (920.49 ± 50.66 mg kg^−1^ and 487.49 ± 20.85 mg kg^−1^, respectively) soil were significantly higher than those in LD (490.41 ± 39.59 mg kg^−1^ and 379.19 ± 9.24 mg kg^−1^), MD (496.25 ± 27.63 mg kg^−1^ and 297.74 ± 28.35 mg kg^−1^) and HD (354.44 ± 1.17 mg kg^−1^ and 286.56 ± 31.66 mg kg^−1^) soils. Moreover, there were significant differences in soil DOC contents between different treatments in the 0–10 cm layer in spring and summer, while no significant differences were found in other layers (P > 0.05). In autumn, there were significant differences in soil DOC contents between 0–10, 20–40, and 40–60 cm layers (P < 0.05), but no significant differences between 10–20, 60–100 cm layers (P > 0.05). In winter, DOC at the 0–10 and 10-20 cm layer for the ND treatment was significantly higher than the other three treatments, while the DOC content of the 20–40 cm layer was lower than the LD treatment, and no significant difference was found between the 40–100 cm layer treatment (P > 0.05). Moreover, the soil average EOC content gradually decreased with the deterioration of vegetation degradation (Fig. 8). The ND plot EOC content in the 0–10 cm layer was significantly higher than other three degradation levels (P < 0.05), while no significant differences were observed in the 10–100 cm layer throughout the year (P > 0.05). The highest mean EOC content was mostly observed during the summer, i.e., 8.32, 5.81, 5.50, and 5.85 g kg^−1^ in ND, LD, MD, and HD, respectively. The soil MBC, DOC, and EOC contents decreased gradually with the increase of soil depth under the four degradation levels. The repeated-measures ANOVA showed significant interactions between the season and vegetation degradation on MBC, DOC, and EOC content in all samples (Table 2).

### Correlations between soil properties and soil C pools

**Figure 9:**
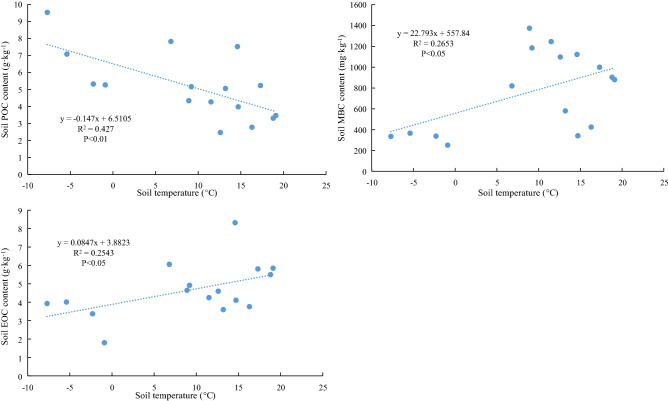
The soil temperature has a significant linear relationship with the content of POC, MBC, and EOC.

We analyzed the correlation between soil characteristics and SOC fractions (Table 3). Significant negative correlations were observed between BD and POC, DOC, MBC, EOC, LFOC, and SOC (R = − 0.915, − 0.850, − 0.984, − 0.799, − 0.799, and − 0.874, respectively, P < 0.01). Soil pH was positively correlated with POC, DOC, MBC, EOC, LFOC, and SOC (respectively R = 0.870, 0.899, 0.656, 0.912, 0.943, and 0.905, P < 0.01 or P < 0.05). Significant positive correlations were detected between TN, vegetation biomass (VB) and SOC components (P < 0.01), while no significant correlation was found between TP and SOC components. Moreover, soil temperature had a negative linear relationship with POC (R^2^ = 0.427, P < 0.01; Fig. 9), and it had positive linear relationship with MBC and EOC (respectively R^2^ = 0.265 and 0.254, P < 0.05).

**Table 3:** Correlation coefficients (r) between soil organic carbon and basic soil properties at a depth of 0–100 cm across the four treatment.

	BD	pH	TN	TP	AB	ST
POC	− 0.915**	0.870**	0.976**	− 0.088	0.965**	− 0.654**
DOC	− 0.850**	0.899**	0.969**	− 0.001	0.952**	− 0.112
MBC	− 0.984**	0.656*	0.867**	− 0.342	0.936**	0.515*
EOC	− 0.799**	0.912**	0.965**	0.071	0.910**	0.504*
LFOC	− 0.799**	0.943**	0.949**	0.153	0.887**	− 0.203
SOC	− 0.874**	0.905**	0.989**	− 0.046	0.954**	− 0.295

*BD* bulk density, *TN* total nitrogen, *TP* total phosphorus, *AB* aboveground biomass, *ST* soil temperature.

*Significant differences at the 0.05 probability level; **Significant differences at the 0.01 probability level.

## Discussion

The soil C pools in wetland systems are considered of great importance in reducing atmospheric C load and mitigating global warming^53^. Compared to other types of wetlands, the SOC content of Gahai wet meadow (located in the southeast edge of QTP) was higher than that of the Chinese delta coastal wetlands^54^ and Hangzhou Bay tidal flat wetlands^21^, lower than that of peatlands^55^, and similar to that of alluvial wet meadows in central Sierra Nevada in the United States^56^. The differences in SOC contents among different types of wetlands are mainly due to the differences in climate, soil type, vegetation composition and environmental factors between locations.

In our study, a significantly higher SOC content was measured in the ND plot than in the other levels of vegetation degradations plots at soil depths of 0–10 and 10–20 cm (Fig. 3). This confirms our first hypothesis that vegetation degradation significantly reduces SOC content in the topsoil of wet meadows and is consistent with previous observation^36,51^. These results may be attributed to the following reasons. On the one hand, the increased intensity of vegetation degradation can increase soil bulk density (Table 3), which will result in changes in soil moisture, air and heat conditions changes, declining soil porosity, and increasingly compacted soil, and thus increase the loss of soil organic carbon content^57^. On the other hand, the decrease of plant biomass in the degraded plots reduced the quantity of carbon resources returned to the soil, resulting in the decrease of soil nutrient content, especially because the maximum grassland rooting depth is 20 cm^58^. Another possible explanation may be that the groundwater level of wetland soil is shallow, which may cause frequent flooding conditions and thus low oxygen content. The oxygen that is present will dissolve and produce a large number of CO_3_^2−^ and HCO_3_^−^ ions, resulting in weak alkaline soil. At the same time, vegetation degradation reduced the pH of the soil, thus increased the activity of soil bacteria^59,60^, and promoted the mineralization and decomposition of SOC. The significant correlations observed between SOC content and BD, pH, TN and aboveground biomass further confirmed our first hypothesis (Table 3). Moreover, the SOC content was higher in spring and winter and lower in summer and autumn, and it mainly concentrated in the top soil layer of 0–20 cm. This is possibly because higher soil temperature and moisture in summer and autumn promote the decomposition of soil C, causing SOC to be emitted into the atmosphere in the form of CO_2_ or CH_4_^61,62^. In spring and winter, the mineralization of SOC is weakened due to lower soil temperature^63^. Furthermore, withered vegetation (litter) increases the amount of carbon resources returned to the soil^64^, leading to the accumulation of SOC.

MBC, DOC, and EOC are important components of soil labile C pools. In this study, the MBC content in the top layer of soil (0–20 cm) in spring and summer was significantly higher in the ND plot than in the other vegetation degradation plots. The reduced litter (Table S1) inputs due to vegetation degradation decreased the MBC content by affecting the energy substrate necessary for the survival of soil microorganisms^65^. Furthermore, the MBC content in the 0–20 cm layer of soil of the ND treatment was the lowest in autumn. We surmised the higher vegetation cover (Table S1) in the ND plot lowered the soil temperature below the vegetation (Fig. 9), resulting in a decrease in soil microbial activity and organic carbon conversion rates^66,67^. Moreover, our results showed that MBC contents of the four vegetation degradation leves were higher in summer and autumn and lower in winter. This may be because the temperature in the study area is higher in summer and autumn (Fig. 9), and plants and microorganisms have entered the peak growth season. More root exudates increase soil microbial activity^68^ and promote the conversion and accumulation of soil active organic carbon. Studies have shown that the content of DOC in soil depends on the input of soluble plant residues and quickly returns to a lower level due to the rapid decomposition of soluble residues^17^. This is similar to our results support those studies because the DOC content in the 0–10 cm layer of ND soil was higher than that of the other treatments, while DOC contents of the deeper soil layers did not significantly differ from that of the other treatments. In addition, the DOC content was lower in summer and autumn and higher in spring and winter because the study area has more rainfall in summer and autumn, which greatly affects the losses of DOC due to leaching^69^. This is consistent with the explanation proposed by Liu et al.^70^ soil DOC content is mainly controlled by precipitation^71^. At the same time, we found that the EOC content decreased significantly with the intensification of vegetation degradation, which is consistent with the findings of Xiao et al.^14^ where more surface biomass (Tables S1 and S3) led to an increase in litters inputs, thereby enriching soil accumulation of EOC. Studies have shown that soil temperature has a great influence on EOC content^72^, which is consistent with our result where a significant positive linear relationship was found between soil temperature and EOC content (Fig. 9). Further, we found that the maximum soil EOC occurred in summer, while the minimum occurred in winter. The main reason is that the higher temperature and sufficient rainfall in summer increases microbial activity^73^ and promotes the decomposition of litter. Additionally, the greater photosynthetic activity in summer increases the release of root exudates^74^. In winter, the drastic drop in soil temperature reduces the MBC content (Fig. 6) and the decomposition rate of litter, resulting in a significant decrease in root exudates and carbohydrates^75,76^. Thus the EOC content reached the lowest level in winter.

The soil carbon fractions LFOC and POC are mainly comprised of litter residues and microbial debris^77,78^. Our results show that the contents of LFOC and POC in the soil surface of the ND treatment were significantly higher than their corresponding contents in the other treatments. These results are likely due to the greater amounts of surface biomass and root exudates in the ND treatment which increases the inputs of soil organic carbon^79,80^. Moreover, we found that the contents of LFOC and POC were greater in spring and winter, and lower in summer and autumn. This is because the soil freeze–thaw cycle that mainly occurs in the soil surface in spring and winter^81^ may have significantly affected the contents of larger soil aggregates^82,83^. Moreover, higher vegetation coverage increases the number of freeze–thaw cycles^84,85^, and lower soil temperature causes microbial cell fragmentation^86^, thereby increasing the sources of LFOC and POC^48^. The negative linear relationship between soil temperature and POC content further confirms this conclusion (Fig. 9). The temperatures in summer and autumn increased the metabolic activity of microorganisms (Fig. 6), and more rainfall increases SOC leaching^87^, resulting in the decreased LFOC and POC contents.

Previous studies have shown that soil organic carbon is mainly derived from the litter of aboveground plant materials and belowground root exudates^88,89^. This is consistent with our research results, which showed that SOC and aboveground plant biomass was significantly positively correlated (Table 3). We also found that soil properties also affect SOC content to varying degrees (Table 3). Because soil active organic carbon is highly sensitive to environmental changes, differences in soil active organic carbon components were observed due to soil environments, nutrients (Table 1), and litter quality/quantity^90–92^. In addition, our results show that the trends in seasonal variation of soil organic carbon components are not consistent with the different vegetation degradation levels, which is further supported by the study carried out by Guo et al.^17^ and Shao et al.^21^. They found that in the same ecosystem (forests and wetlands), the trends in seasonal variation of different active organic carbon components are not entirely consistent because the seasonal variation of soil active organic carbon content is a complex process of interaction of multiple factors^76^. Therefore, the Gahai wet meadow exhibited significant seasonal variation in the content of soil active organic carbon due to its vegetation composition, litter quality/quantity, and soil properties (Tables S1, 1 and 2), such as soil BD, pH, soil temperature, and nutrient input^25,93–95^.

## Conclusion

Vegetation degradation significantly affected the vertical and seasonal distribution of SOC and its different components (MBC, DOC, EOC, POC, and LFOC). Among the four vegetation degradation levels, the ND plot had a significantly higher SOC content throughout the year. In addition, the greatest differences in SOC were observed in surface soil, and considerable variations were also observed in deeper soil layers. The results suggest that vegetation degradation reduces the accumulation of total SOC and its different components, which may reduce the ecosystem function of C sequestration for the alpine wet meadow, and thus reduce soil quality. Moreover, we found that there is inconsistency in the seasonal variation of the different components of SOC. Pearson correlation analysis showed that soil properties and unstable carbon components played a key role in the formation of soil C. In addition, high altitude wet meadow is one of the most sensitive ecosystems to climate change, especially vulnerable to climate warming. The potential activities of different components of SOC will activate and change the biogeochemical cycle of carbon and nitrogen in these wetlands. This study not only contribute to evaluate the dynamics of soil carbon in high-altitude wetland under the background of global warming, but also provides theoretical basis and scientific support for QTP alpine degraded wetland ecosystem to regulate the stability of soil organic carbon components and enhance its carbon sink function. Although the sampling time of this study was limited, this study showed that the soil carbon pool in a wet meadow depends on season and soil depth, and encourages further research using a longer sampling time and microbial community structure to refine our understanding of how vegetation degradation affects the ecosystem function of alpine wet meadows.

## Supplementary Information

Supplementary Information.
