# Supplementary material for: Vertical and seasonal changes in soil carbon pools to vegetation degradation in a wet meadow on the Qinghai-Tibet Plateau
Source: Sci Rep. 2021 Jun 10;11:12268. doi: 10.1038/s41598-021-90543-6 (PMC8192520; doi:10.1038/s41598-021-90543-6)
Supplement: Supplementary file 1 — Supplementary Information. [file 41598_2021_90543_MOESM1_ESM.doc]

**Vertical and seasonal changes in soil carbon pools to vegetation degradation in a wet meadow on the Qinghai-Tibet Plateau**

Jiangqi Wu^1a^, Haiyan Wang^1a^, Guang Li^*a^, Jianghua Wu^b^, Weiwei Ma^a^

^a^College of Forestry, Gansu Agricultural University, Lanzhou, 730070, China.

^b^School of Science and the Environment, Memorial University of Newfoundland, 20 University Drive, Corner Brook, NL, A2H 5G4, Canada.

*Corresponding Author: Guang Li

e-mail: liggsau@foxmail.com

Table S1. Basic situation of the plots

| Degradaation degree | Elevation/m | Vegetation cover/% | Biomass/g·m^-2^ | Basic information |
| --- | --- | --- | --- | --- |
| ND | 3477 | >90 | 378.40 | Wet plants are the dominant species, with more litter and root systems, shallower seasonal water, and groundwater level of 20-40 cm. |
| LD | 3478 | 75-90 | 308.07 | Wet plants are the main associated species; the exposed surface area is 5% to 10% without water accumulation; groundwater level is 40-70 cm |
| MD | 3484 | 30-70 | 261.21 | Wet plants are common companion species or occasional species, and some poisonous weeds appear; the exposed surface area is 10% to 30% without water accumulation; the groundwater leve is below 70 cm. |
| HD | 3486 | <10 | The vegetation is sparse, with only sporadic droughts; the exposed surface area is over 90%. | |

ND, non-degraded; LD, lightly degraded; MD, moderately degraded; HD, heavily degraded.


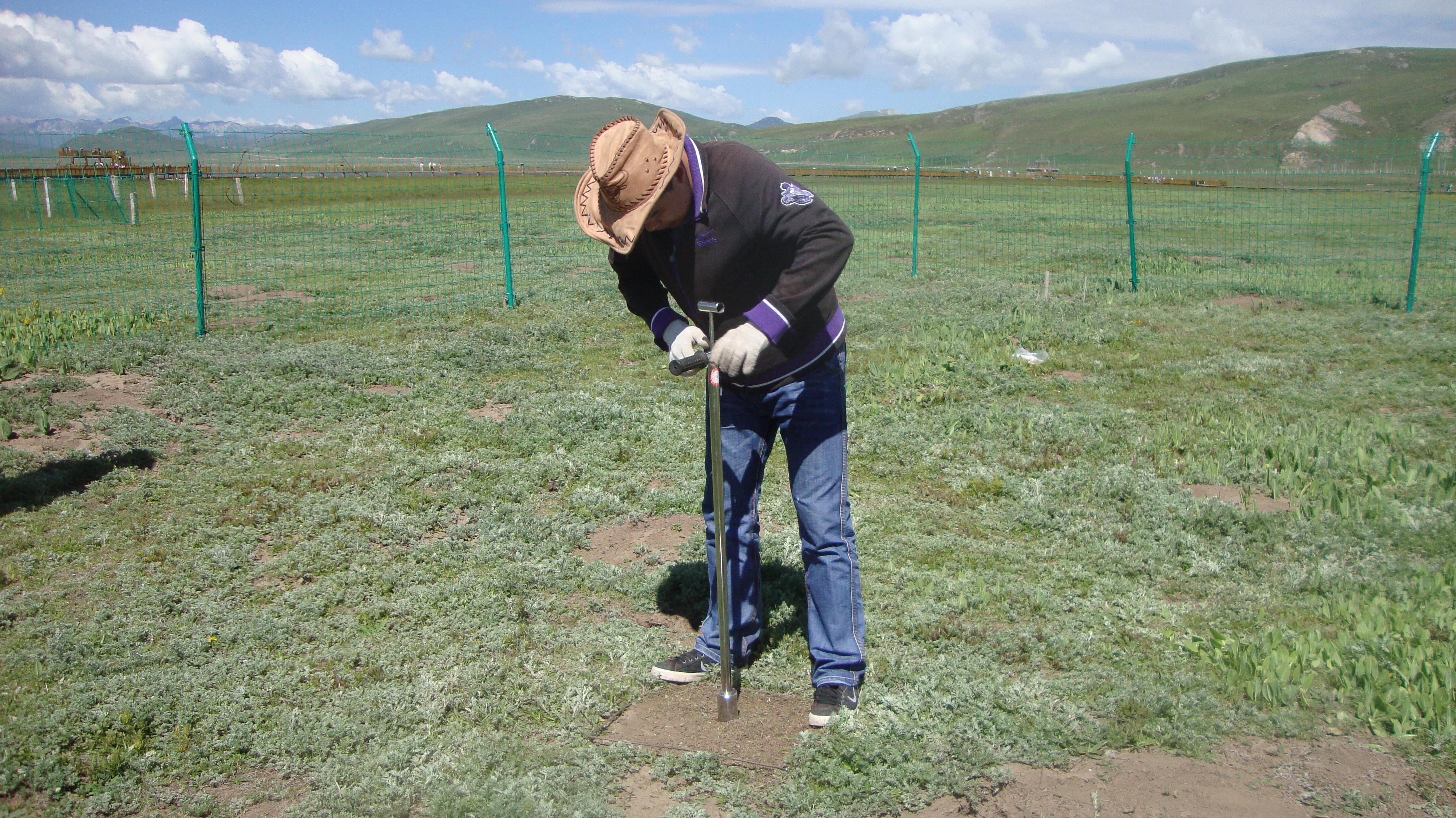


Fig S1 Soil photos collected on the sample plot.


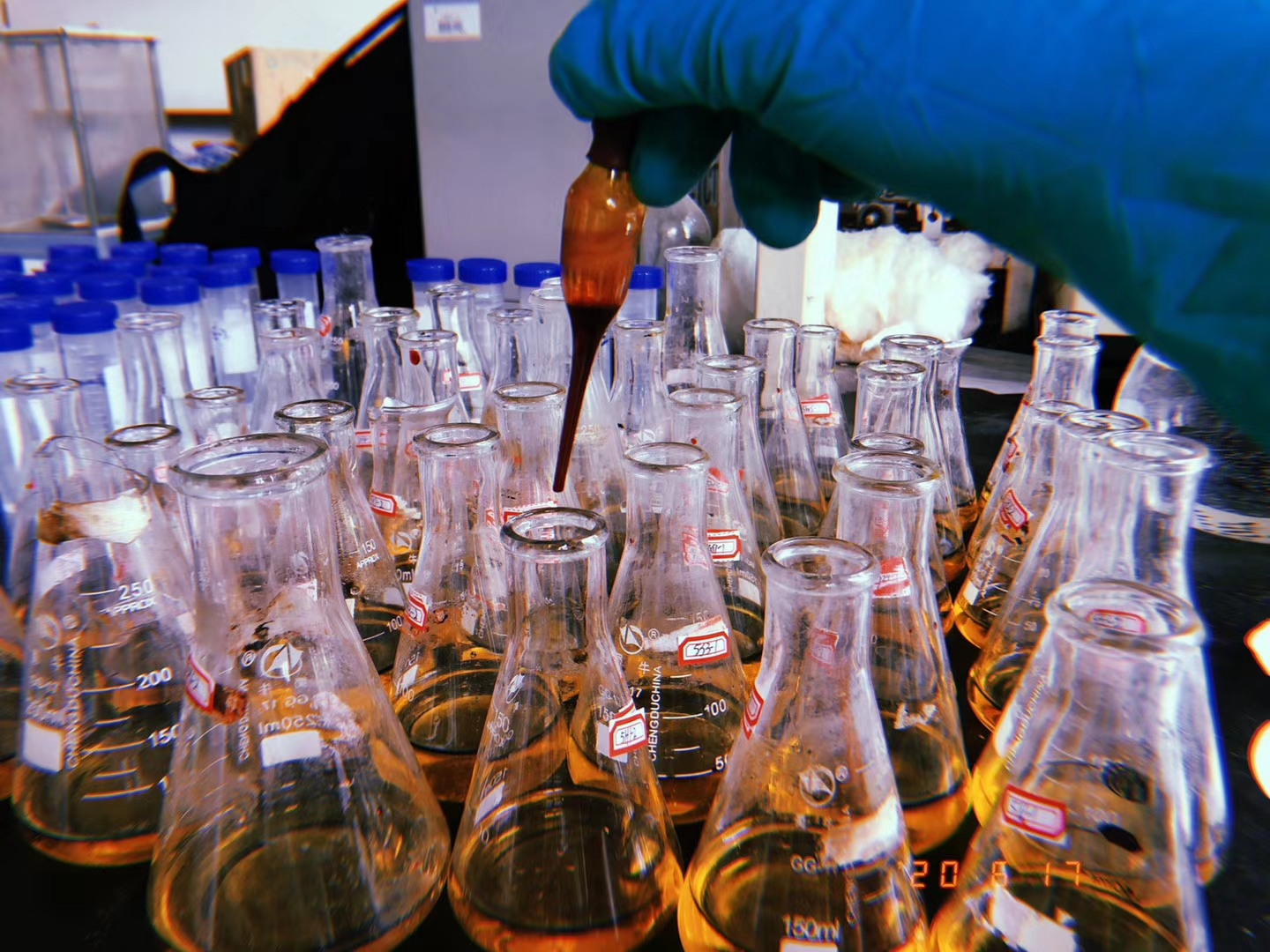


Fig S2 Photographs of soil samples measured in the laboratory.
